# Application of Cognitive System Model and Gestalt Psychology in Residential Healthy Environment Design

**DOI:** 10.1155/2022/5661221

**Published:** 2022-08-21

**Authors:** Jicheng Yang, Chao Yuan

**Affiliations:** ^1^School of Design, Beijing Normal University, Zhuhai, Guangdong 519087, China; ^2^College of Economics and Management, Nanjing University of Aeronautics and Astronautics, Nanjing, Jiangsu 210016, China; ^3^School of Design, Jiangnan University, Wuxi, Jiangsu 214122, China

## Abstract

The influence of the environment on people is very large. According to the general rules of people's daily routines, people spend at least half of their time in residential areas. Therefore, the environment of residential areas has a great impact on people's emotions, behaviors, and even the development of living habits. Residential area refers to a residential area where residents live in clusters and form a certain scale, including buildings where people live, public buildings and facilities for rest, education, fitness, work, and even communication between people, green space, and traffic roads. Usually, the environmental design of urban residential areas usually meets the requirements of diverse functions, strong compatibility, and convenient travel, so as to facilitate residents' living. In an ideal state, the environmental design of a residential area should not only meet the basic living conditions but also improve the living comfort of the residents. Because there is a close relationship between people's psychology and behavior and the living environment of the community, environmental design that is in line with people's positive and optimistic psychology helps residents maintain a happy mood. Therefore, from the perspective of environment to residents' living comfort, this paper introduces a cognitive system model and Gestalt psychology to optimize the design of residential healthy environment. A more harmonious and comfortable living environment is established by using the principle of bottom, grouping, and simplification in Gestalt.

## 1. Introduction

The living environment refers to the accommodation within the scope of the residential area, the surrounding things related to the living behavior, and the corresponding psychological feelings generated by people. The residential area environment includes both the tangible environment, that is, natural elements, artificial elements, and social elements. Its specific performance is the space formed by the combination of various entities, including residential buildings, public service facilities, structures, roads, squares, green spaces, and various activity places, which together constitute the physical environment of the residential area. The living environment also includes intangible and spiritual things. For example, residents' interest in life, information exchange and communication, social order, neighbor relationship, spiritual outlook, moral cultivation, customs, security, and sense of belonging. The improvement of people's living standards makes modern residents have higher requirements for the living environment. From the beginning, people only need a comfortable house, and now they are pursuing a better and better external environment. Based on this, the environmental design of the residential area began to enter people's sight and attract people's attention. From a macro perspective, the environmental design of the residential area is a part of the urban and even regional environmental design. Therefore, it is closely related to the development of cities, the changes in urban life, and the protection and optimization of natural ecology. From a microscopic point of view, the environmental design of residential areas serves people, and is closely related to people's behaviors and psychological needs. The environmental landscape of the residential area is not only for people to see but also for people to use.

The domestic environmental design of residential areas has risen from the traditional material level design to the spiritual level. The high-quality residential healthy environment design can make the occupants feel comfortable and satisfied psychologically. The relationship between the living environment and people's psychology has become a problem that scholars are very concerned about. Therefore, the discipline of environmental psychology was also born one after another [1, 2]. Reference [3] introduces environmental psychology into the design of buildings. This study shows that the control of spatial communication in architectural design leads to the control of behavioral communication in interpersonal communication is very important. Reference [4] introduces environmental psychology and social psychology into all aspects of human life and discusses their impact on people's well-being. Reference [5] explores the relationship between man and architecture, nature, and living environment. The study pointed out that the purpose of environmental psychology is to explore the relationship between man and nature. Reference [6] pointed out that environmental psychology should give priority to the reciprocal relationship between people and the environment. Reference [7] introduces psychology into social environment and life. Reference [8] presents a comprehensive framework for promoting healthy habits based on environmental psychology. Reference [9] discusses the relationship between feng shui and psychology, and studies the influence of feng shui on people's lives. Reference [10] assesses how the built environment is influenced by human life as well as the urban landscape. The above research studies the relationship between the environment and psychology from the perspective of psychology, and proves that people's psychological activities are affected by the environment.

It is based on the above research conclusions, in order to improve the environmental comfort of the residential area, this paper introduces the psychological theory into the environmental design of the residential area. Typical psychological theories mainly include cognitive system model and Gestalt psychology [11–13], color psychology [14–16], cognitive psychology [17–19], ecological self-consciousness theory [20], and probability self-consciousness theory [21]. Gestalt psychology is most widely and successfully used in interaction design. Therefore, this paper applies the cognitive system model and Gestalt psychology to the environmental design of residential areas. Gestalt principles of organization theoretically clarify the relationship between perceptual integrity and form. In the residential healthy environment design, the use of Gestalt's organizational principles can increase the richness and observability of the landscape, and it is convenient for residents to increase their awareness and understanding of the landscape from the perspective of formal beauty.

## 2. Related Concepts

### 2.1. Introduction to Residential Area and Environment

**Figure 1:**
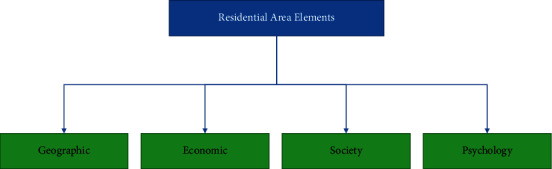
Composition of residential area.

The characteristic of the residential area is that the residences are concentrated, and there are a certain number and corresponding scale of public service facilities. It can provide residents with housing, recreation, and daily life services. Residential areas have a specific scale in urban planning. Usually the population is about 40,000 people, and the land area is about 80 hectares. The components of the residential area are shown in Figure 1:

The environment of the residential area refers to the living place of the residents, the surrounding things related to the behavior and the corresponding psychological cognition generated by the people within the scope of the residential area. The elements of residential area mainly include geographical elements, economic elements, social elements, and psychological elements. Geographical elements mainly refer to the physical environment such as buildings, public facilities, green plants, roads. Economic factors refer to shopping and consumption in the community. The social element refers to the social interaction between residents such as the interaction between neighbors. Psychological elements refer to the overall division of the residential area presented by the environment of the residential area such as the mood of the residents, the relationship between neighbors, and the spiritual outlook.

### 2.2. The Composition of the Environmental Landscape of the Residential Area

The living environment can be divided into different types according to different standards. One divides the living environment into material space environment and nonmaterial space environment, and the other divides the living environment into outdoor environment and indoor environment.

The physical space environment refers to the basic needs environment on which people live, including buildings and engineering equipment such as housing, squares, green space, commercial, service, and cultural facilities. A good material space environment has good air quality, sufficient sunlight, water environment, and green space for plants, as well as perfect and convenient facilities. These can meet people's living needs and behavioral needs. The material space environment design not only includes the buildings and greening of the residential area but also includes green space decoration, walls, gates, activity facilities, various signs, water features, sculptures, lighting facilities, audio facilities, etc. These contents must be organically integrated with the residential building to form a harmonious whole.

**Figure 2:**
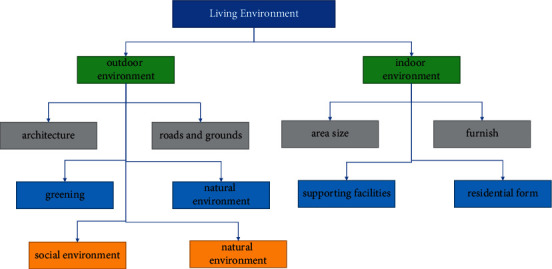
Composition of indoor and outdoor environments.

The immaterial space environment is reflected in spiritual, informational, and psychological belonging. It mainly includes mental space, information space, and psychological space. Spiritual space can bring people beautiful sentiments and creative thinking ability. Information space can shorten the distance between people and enable information sharing. Mental space is built on people's concept of home. Home is a shelter for people both physically and psychologically. Nowadays, many communities build a sense of community identity and build themed homes from this point. The components of the outdoor environment and the indoor environment are shown in Figure 2:

Through the classification of the environmental landscape of the residential area, it can be concluded that the constituent elements of the residential area landscape can be divided into natural elements, artificial elements, and social elements. Natural elements mainly include native landscapes such as hills, woods, and lakes. Artificial elements mainly include rockeries, flower beds, fountains, statues, etc., in the residential area. The social elements mainly include activities held in residential areas and places for leisure and entertainment.

### 2.3. Cognitive System Model and Gestalt Psychology Theory

**Figure 3:**
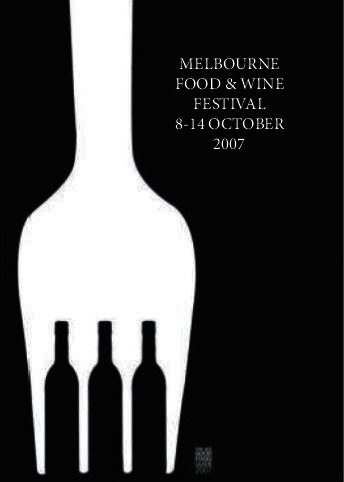
Principles of map and map.

In 1912, a German scholar put forward the theory of Gestalt psychology. Gestalt psychology mainly contains two meanings: one refers to the general attribute of the transaction, namely the form; the other refers to the individual attribute of the transaction, that is, the separated whole, and the form is only one of its attributes. This theory mainly emphasizes the relationship between the whole and the parts of things. A whole may be composed of multiple parts, and the parts are combined to form a whole. And the reason why each of these parts play their own role is because they do not exist alone, but in the whole. Gestalt psychology does not look at things from separate parts, but studies problems as a whole. In daily life, when people perceive an object, they like to organize and order the object, so that it is easier to enhance the understanding and adaptation of the object. In Gestalt psychology, there are three main organizational principles, namely, the principle of map-ground relationship, the principle of grouping, and the principle of simplification. The map-ground relationship means that when people observe an object, they cannot fully perceive all the information of the image, but can only perceive part of the information. The perceived information forms the image, and the unperceived information is called the background. The schematic diagram of the map principle is shown in Figure 3.

**Figure 4:**
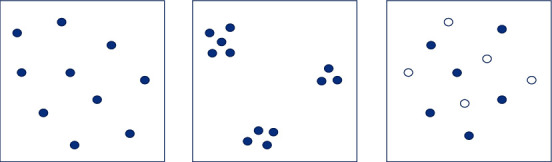
Grouping principle.

When we observe things, Gestalt psychology believes that human perception can link similar elements together so that they form an organic whole. This law of perceiving similar elements as a unified whole is called the grouping principle. The grouping principle is shown in Figure 4.

**Figure 5:**
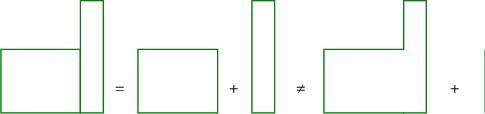
Simplification principle.

Gestalt psychology believes that when people perceive graphics, in order to be more conducive to their own understanding and cognition, they usually prefer to simplify operations on graphics, which is called the principle of simplification. The simplification principle is shown in Figure 5.

## 3. The Relationship between Residents and the Environment in Which They Live

### 3.1. The Relationship between Residents and the Environment of the place of Residence

The living environment can be divided into two parts: hard environment and soft environment. The hard environment refers to the hardware facilities of the community, such as fitness facilities, activity rooms. Soft environment refers to the cultural literacy, civility and politeness of community living. The two must be interdependent and constrained. A good living environment shows that the soft environment and the hard environment complement each other and promote each other. A bad living environment either has only one aspect, or even lacks both aspects. At present, the hard environment of each residence has reached the basic needs of residents, but the soft environment needs to be further improved. There is a close relationship between the living behavior of residents and the living environment. The construction of the soft environment in the residential area mainly depends on the behavior of the residents. People-oriented is a common requirement for the upgrading of contemporary living environment. Human life needs are divided into material needs and spiritual needs. Living needs are embodied in various activities of residents, which are divided into routine activities, occasional activities, and social activities. Each activity has different requirements for the environment.

Routine activities refer to the activities that residents need to complete every day, such as commuting to get off work, commuting to school, grocery shopping, cooking, washing dishes, mopping the floor. These are activities that must be done on a daily basis. These activities are seriously affected by the environment of the residential area, especially the effect of the material environment. For example, whether the public facilities in the residential area are complete, and whether the transportation is convenient. Whether it is convenient to pick up and drop off children on the way to and from get off work, whether it is convenient to shop and park vehicles, etc. Once these physical environments cannot meet the conditions for engaging in essential activities, it means that the residential area does not have convenience and safety. Accidental activities are activities that only occur under the right circumstances. In contrast to routine activities, it occurs only when people have the will and when and where it is permitted. Such activities include mountain climbing, running, and watching movies. The frequent occurrence of contingent activities is an important criterion to test whether the environmental planning is in place. For example, the greening of the community is well done, and the active atmosphere of the running track and promenade design can stimulate people's willingness to exercise. Social activities are behaviors that require the participation of other people. Such as marathon, community encyclopedia knowledge contest.

To sum up, a good residential healthy environment can not only satisfy people's regular activities, but also promote residents' contingency and social activities. So as to promote interpersonal communication among residents, fitness exercise. The development of outdoor activities among residents also enhances the soft environment of the residential area, reflecting that the residential area environment is full of vitality and the neighborhood relationship is good. By analyzing the relationship between the residents' behavior and the environment of the residential area, it is concluded that a good residential healthy environment can promote the residents to have a sense of intimacy and dependence on the living environment. And this generalization of intimacy and dependence leads to a sense of belonging to the environment. For residents, there are many factors that affect the sense of belonging, such as the sense of security, comfort, and privacy mentioned in the ecological perception theory. In the design of the residential landscape environment, we must pay great attention to these psychological needs of people.

### 3.2. Residents' Psychological Needs and Residential Healthy Environment Design

For the environment of the residential area, due to the different ages, personalities, and hobbies of the residents, the requirements for environmental design are also different. According to age, residents can be roughly divided into the elderly, young and middle-aged, and children.

The elderly pay more attention to the comfort and leisure of the body, and require more interest in life, so as to pass the time. The elderly pay more attention to the accessibility of communication, and at the same time have higher requirements for safety and convenience. Therefore, in the construction of the garden landscape design in the residential area, a special activity place should be opened up for the elderly. This is not only convenient for the elderly to exercise and exercise but also a platform for the elderly to communicate with each other. Thereby it can ensure the fun of communication for the elderly and satisfy the emotional lack of psychological aspects.

For young and middle-aged groups, they pay more attention to the quality of the environment and the design of ecological green plants, because a comfortable and green environment can make people more physically and mentally relaxed. Based on this demand, first of all, we should increase the coverage of green plants as much as possible, and set up some leisure places such as coffee houses, reading bars, which can enable young people to communicate better. Secondly, young and middle-aged groups like to exercise, and some running tracks should be set up in the residential area as much as possible to facilitate walking and running, and there should be no shortage of sports venues, such as table tennis tables or basketball courts. In this way, young and middle-aged groups can smoothly carry out sports and exercise in the residential area. It should be noted here that sports venues should maintain a certain distance from residential buildings so as not to affect the rest of residents. Because different groups of people have different time periods for rest and exercise, some people like to get up early to exercise, and some people like to exercise in the evening. Not disturbing the daily life of other residents is the most basic requirement.

For groups of children, they mainly focus on entertainment and fun in the residential area. For example, for the design of garden landscapes, children like the bright colors of the vegetation, the contrast is large, the shapes are varied, interesting, and lovely. In the rest area of the community, on both sides of the promenade, and in the children's playground, some cartoon and animation elements can be set up to increase the fun of the place. Second, child-optimized places are a top priority for groups of children. First of all, openness should be considered, because usually young children are accompanied by adults to play. The place is too small to fit, the place is too closed and uncomfortable. Most children like to slide and play with sand. When purchasing amusement equipment, there should be a priority. Children also like to be close to nature. For garden trees, you can plant some tree species with bright flowers or leaves and seasonal changes to enhance the ornamental effect of the trees and increase the interest of the garden. These improvement measures can give full play to the attractiveness of residential gardens to children.

In summary, we can conclude the following rules. First, no matter what type of people, there is a demand for green plants. The general requirements for green plant design are bright colors and interesting shapes. The second is to have suitable sports venues. In addition to considering residents' preferences for sports, outdoor sports should also be taken into account. Outdoor exercise can ensure enough sunlight, which is very important for children and the elderly. Proper exposure to sunlight can supplement calcium and is good for physical and mental health. The third is to have leisure and entertainment venues. The purpose of this place is to facilitate communication between residents. Therefore, a casual, comfortable, slow-paced atmosphere should be created.

## 4. Residential Healthy Environment Design Based on Cognitive System Model and Gestalt Psychology

### 4.1. Interior Design

**Figure 6:**
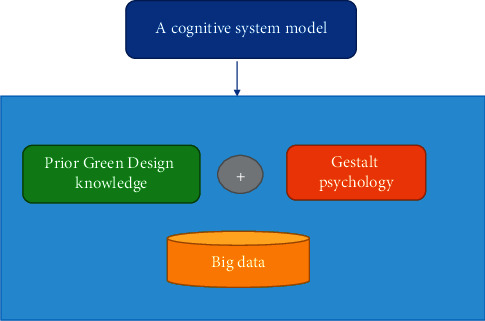
A cognitive system model for healthy environment design.

**Figure 7:**
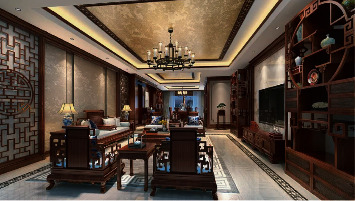
Chinese style interior design.

The overall theory is that the components of the indoor space have different styles, but they tend to be consistent as a whole. Cognitive system model and Gestalt psychology believes that perception is not the addition of multiple individual elements, nor is it a specific element, but the perception of the whole (See Figure 6). That is to say, the whole is greater than the sum of its parts. The interior space design can use different materials and different styles of decorations to match together, and finally let people perceive an overall decoration style. Gestalt psychology believes that the addition of parts does not equal the whole, and the whole is greater than the sum of the parts. The combination of psychological field and physical field enables us to perceive more connotations than things themselves, which is also the “design style” that interior designers have been trying to create. For example, Chinese style interior design is mainly reflected in traditional furniture, decorations, and black and red-based decorative colors. Symmetrical layouts are often used in the interior. The style is elegant, the shape is simple and beautiful, and the color is strong and mature. Indoors generally have calligraphy and painting, plaques, hanging screens, bonsai, porcelain, antiques, screens, Bogu frames, and so on. The Chinese style is shown in Figure 7.

Gestalt psychologists believe that the observer's perception is a “field” concept. The mind-object field is the combination of the psychological field and the physical field. The identity, status, and age of the space users are considered in the design, and the physical field is designed according to different psychological needs. Due to the theoretical basis of isotype theory, the psychological field and the physical field will correspond one by one, and finally create an interior space that makes users feel warm, which is also the essence of interior design.

There are many “physical fields” in interior design, such as materials, lighting, and functions. Different “psychological fields” will be directly reflected in “physical fields”. For example, the interior design and decoration of kindergartens are different from ordinary home decoration. The choice of color system should be based on the physical and psychological needs of children. The right color is in line with the growth process of children. To create a healthy learning and living environment for them, cool colors should be used carefully, and warm colors can bring a warm home-like feeling to children. For example, green will bring imaginative space to children, and the decoration of kindergarten should be combined with the region and the selected theme to determine the decoration style. A kindergarten uses orange, pink, yellow, green, purple, blue, and other different colors as the theme colors of each classroom, including wallpaper, table, and chair colors. This is to take full advantage of the influence of different colors on mental activity. Therefore, if the physical field is designed according to specific psychological needs, it can fill our indoor space with “temperature” and make people feel more comfortable.

### 4.2. Outdoor Environment Design

**Figure 8:**
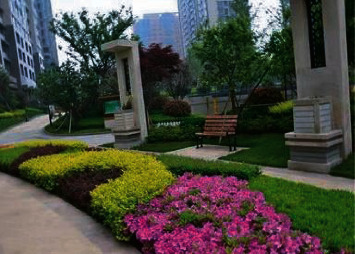
Green plant design.

Outdoor environment design mainly includes ecological landscape design, activity place design, rest place design, and public space design. Ecological landscape design is mainly divided into green design, water environment design such as fountains and pools. For the greening design, it is mainly designed from the arrangement, shape, height, and size of the plants. Based on the theory of Gestalt psychology, it is known that the design of green plants needs to avoid a single color tone, and the combination of plants with contrasting colors will be more eye-catching. An example of the design of green plants is shown in Figure 8.

**Figure 9:**
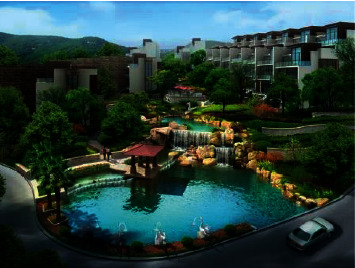
Water environment design.

One visual perception forms a figure, and it is highlighted on another background figure, this phenomenon is one of the basic principles of Gestalt graphics principle. The relationship between “shape” and “bottom” is dialectical, they are interrelated and interdependent. In the visual realm nothing is negative, positive can be negative and negative can be positive. Therefore, it is necessary to make overall plans and make full use of the changing relationship between “shape” and “bottom” in the design to obtain a perfect and interesting visual effect. In the community, residential buildings are regular, small in size, with relatively clear outlines, and are in a dominant position. It has a great influence on the layout and landscape design of the whole community. Therefore, designers often regard residential buildings as “pictures” and green landscapes as “bottoms”, in order to more clearly distinguish the relationship between the picture and the bottom in the community.  Figure 9depicts a schematic diagram of the community's artificial pool. The pool is placed not far from the entrance of the community. The reflection of the blue sky and white clouds on the water surface, as well as the surrounding buildings and green plants, makes the courtyard present a colorful scene and enhances the integrity of the community. This wholeness makes the courtyard itself take on the character of a “picture”. The arrangement of the buildings forms the “bottom”.

### 4.3. Case Analysis

#### 4.3.1. Residential Area Description

**Figure 10:**
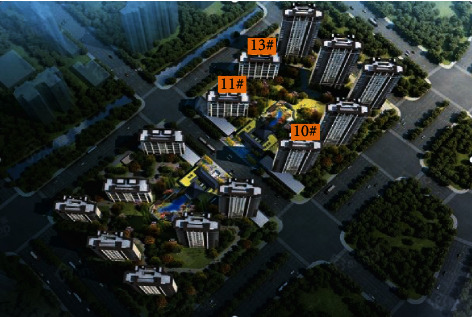
A bird's-eye view of the community.

In a community in an economic development zone, 47% of the residents are young people, 26% are the elderly, and 27% are children. There are many young people in the residents of this community, and they are stingy and full of vigor and vitality on the whole. The community is far from the city and close to the suburbs. The air is fresh and the vegetation is abundant. The area outside the community is wide, but public transportation is not very developed. There are schools and parks within 3 km. The community is rectangular and covers an area of 55,997 square meters. An aerial view of the community is shown in Figure 10. The gate of the community is set in the middle of two square grids, and a road divides the community into two parts.

#### 4.3.2. Environmental Design

Some attractive landscapes should be set up at the entrance of the community to make people feel happy as soon as they enter the community. In addition, more than one small square should be set up in the community to facilitate residents' communication and sunbathing. The design of the small square should be based on the principles of Gestalt psychology, with the surrounding green plants as the bottom and the square in the middle as the map. In the design of small roads in the community, there are various ground paving forms, including regular and rigorous paving forms, as well as irregular and interesting paving forms. In view of the youthful characteristics of this community, some irregular and clustered floor tiles can be laid. In the design of sitting and resting space, the number of seats in the community is relatively large. The basic seats are arranged near the pedestrian path, which is convenient for many residents with limited mobility or physical fatigue to sit at any time. Other auxiliary seats such as steps, low walls, flower terraces, etc., are combined with landscape design to provide services for residents who use it occasionally.

Due to the large number of children in the community, the design of the children's play area is very important. The playground of the community is placed on the side of the main pedestrian street. Through game activities, residents can be attracted to stop and watch, and the vitality and vitality of the street can be increased. Dense plants are planted between the site and the road, which not only prevents children from accidently rushing into the main road but also forms a good boundary, increasing the layering and richness of the space. There are seats for parents of young children to rest around the playground. The floor coverings come in a variety of colors. In the community, the elderly activity facilities are mainly distributed in the form of dots. These facilities include chess and card tables and chairs, fitness equipment, etc. This distribution pattern is conducive to the spontaneous activities of the elderly. For example, a few neighbors and friends who live nearby can easily reach the activity area and play chess and card leisure activities or exercise in good weather. In the community, because there are many seats, it is convenient for the elderly to do it at any time. Through the combination of seats, pavilions, corridors, and other facilities, the elderly are given more sitting and leisure space, which basically meets the needs of the elderly. The various needs of the elderly to sit, lie down, stop, and stay.

## 5. Conclusion

With the development of social economy, residents' demands for the environment of residential areas are increasing day by day. For modern residents, they not only require the living environment of the community to be comfortable and clean but also require that the living environment can give people a warm and decompressed feeling from the soul. This demand has a qualitative change, which highlights the effect of environmental design on the psychology of residents. Inspired by this, in order to qualitatively improve the residential healthy environment, this paper introduces cognitive system model and Gestalt psychology, a typical theory of psychology, into the design of the residential healthy environment. This paper first expounds the definition of residential healthy environment and Gestalt psychological theory. Second, it analyzes the relationship between psychological changes and environmental design, including how the environment affects residents' psychology and how residents' behavior affects the environment. Finally, based on the Gestalt theory, the design suggestions for the residential area environment are given, respectively. Including interior design, ecological green plant design, water environment design. In the design of each module, relevant theories in Gestalt psychology are integrated. The effectiveness of the proposed design method is also demonstrated by relevant examples. However, there are still some problems in this paper that need to be further optimized. For example, the environmental design of the residential area is not complete, including the lighting, leisure space, and sports venues of the residential area. In addition, some other psychological theories are also considered to be introduced into the residential healthy environment design, such as color psychology, cognitive psychology, and so on. This study will carry out in-depth research on these aspects in the follow-up work.

## Data Availability

The labeled dataset used to support the findings of this study are available from the corresponding author upon request.
